# The Evolution of Visual Roles – Ancient Vision Versus Object Vision

**DOI:** 10.3389/fnana.2022.789375

**Published:** 2022-02-09

**Authors:** Dan-Eric Nilsson

**Affiliations:** Lund Vision Group, Department of Biology, Lund University, Lund, Sweden

**Keywords:** vision, visual role, evolution, visual processing, behavior

## Abstract

Just like other complex biological features, image vision (multi-pixel light sensing) did not evolve suddenly. Animal visual systems have a long prehistory of non-imaging light sensitivity. The first spatial vision was likely very crude with only few pixels, and evolved to improve orientation behaviors previously supported by single-channel directional photoreception. The origin of image vision was simply a switch from single to multiple spatial channels, which improved the behaviors for finding a suitable habitat and position itself within it. Orientation based on spatial vision obviously involves active guidance of behaviors but, by necessity, also assessment of habitat suitability and environmental conditions. These conditions are crucial for deciding when to forage, reproduce, seek shelter, rest, etc. When spatial resolution became good enough to see other animals and interact with them, a whole range of new visual roles emerged: pursuit, escape, communication and other interactions. All these new visual roles require entirely new types of visual processing. Objects needed to be separated from the background, identified and classified to make the correct choice of interaction. Object detection and identification can be used actively to guide behaviors but of course also to assess the over-all situation. Visual roles can thus be classified as either ancient non-object-based tasks, or object vision. Each of these two categories can also be further divided into active visual tasks and visual assessment tasks. This generates four major categories of vision into which I propose that all visual roles can be categorized.

## Introduction

Imagine you have just bought a sandwich and a paper mug of coffee and are looking for a place to sit and have your lunch. Across the park you see an unoccupied bench in an attractive spot. To get there, you first follow a paved path and then make a shortcut across the lawn. You sit down at the bench and unwrap your sandwich. You remove a piece of lettuce that does not look fresh before you take the first bite.

In this little glimpse of everyday life, you have relied on vision for a range of rather different tasks. You have assessed your environment to find a suitable place and then used visual input to guide your movement along a path and in the direction to a desired place. You have also assessed objects and used vision to guide manipulation of them. As human-oriented as this example is, we still share the very same general roles of vision with other vertebrates, with numerous arthropods, and with cephalopods. Animals of other phyla have less advanced visual systems serving fewer basic types of roles. There are in fact animals that live and prosper with every imaginable intermediate from simple non-visual photoreception to the full set of visual roles found in vertebrates, arthropods and cephalopods. This makes it possible to reconstruct the evolution of vision from the very simplest forms to the most advanced. Here I will argue that even though vision has evolved many times independently from non-visual photoreception, new roles of vision have, with only few exceptions, been acquired in the same general sequence in all animal groups that have any kind of eyes or vision.

## Setting the Stage for Evolution of Vision

As has been argued elsewhere (Nilsson, 2009, 2013, 2020, 2021), the first opsin-based photoreception was likely used to monitor the daily light cycle, to help choose the right activity at the right time. Given the slow changes in daily light levels, the photoreceptors tracking this intensity change would have been slow and non-directional. Much faster intensity changes are caused by the animal moving in or out of shade or into deeper or shallower water, or by clouds. To adapt the behavior to these faster changes, some photoreceptors may have adopted new roles by speeding up their responses or changing their spectral sensitivity. Knowing about intensity changes caused by moving into differently lit parts of the habitat is useful in itself but will also help separate these signals from that of the daily light cycle. Another way to isolate signals arising from the daily light cycle from those arising from natural light disturbances is to employ a biological clock with photoreceptor input (Brown et al., 2014).

Faster photoreceptors also open possibilities for direct control of locomotion, especially if they become directional through an association with screening pigments. Such photoreceptors can actively steer the animal toward brighter or dimmer parts of the environment, and thus be used to seek out suitable habitats or move to optimal positions within a habitat. Positive or negative phototaxis served by a single directional photoreceptor is a common feature of invertebrates, both in adults and larvae (Randel and Jekely, 2016), and would have been a powerful addition to non-directional photoreception in early metazoan evolution. However, even though light-intensity monitoring and directional photoreception have critical and important biological functions, these behaviors can be mediated by a single isolated photoreceptor, and thus do not result from true vision. Instead, true vision relies on the comparison of signals arising from two or more photoreceptors receiving light from different directions.

## True Vision for Habitat Orientation

There are two principal limitations to directional photoreception. One is that the animal must turn or scan to find the direction of light or darkness. The other is that it allows orientation only to the overall distribution of light – not to the distribution of spatial structures. Simultaneous comparison of signals from several directional photoreceptors aimed in different directions will remove both limitations and provide true vision (simultaneous spatial resolution). Apart from the obvious multiplication of directional photoreceptors, true vision also requires novel neural circuits for discrimination of spatial intensity differences (contrasts) and their motion. With these in place, even a small number of photoreceptors, and thus a very low resolution, opens for much more efficient orientation in the environment. Specific habitats and locations within habitats can be identified, and vision can actively guide locomotion accordingly (Nilsson, 2013, 2020; Randel and Jekely, 2016).

**FIGURE 1:**
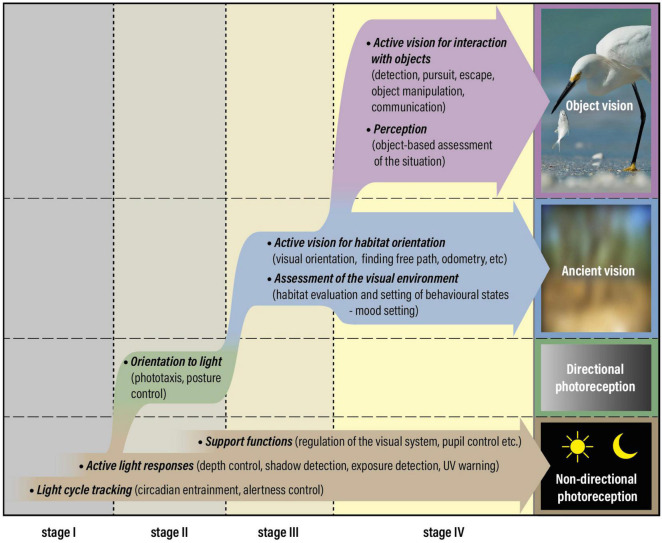
Photoreception and vision have evolved through four distinct stages, starting with *non-directional photoreception* at stage I, acquisition of *directional photoreception* at stage II, which is replaced by *ancient vision* at stage III and complemented by *object vision* at stage IV. True vision and eyes are associated with the introduction of stage III. The figure was originally prepared for a recent review (Nilsson, 2021) but has been adapted to the new concepts of ancient vision and object vision argued for in this manuscript. Ancient vision was not reduced after the introduction of object vision but could massively boost its performance without much additional cost. Consequently, between stages III and IV, vision changed from a minor to a major sensory modality.

Interestingly, true spatial vision replaces directional photoreception, but non-directional photoreception remains as important as before vision evolved (Figure 1). It may even acquire new roles such as controlling light-dark adaptation of visual photoreceptors (Aranda and Schmidt, 2020). There is an important distinction between the information used by visual photoreceptors and that used for determining the time of day or depth in water (irradiance detectors): visual photoreceptors compare relative intensities within a scene, whereas irradiance detectors measure the absolute light level. For visual photoreceptors, changes in irradiance are a nuisance calling for adaptation mechanisms that dynamically change the gain to match the current irradiance (note that the difference between the darkest and brightest parts of a single scene is 1–2 log units, whereas the daily light cycle covers 8 log units). Consequently, visual photoreception and irradiance monitoring require very different photoreceptor properties. Irradiance detection to support vision may thus be a reason for co-expression of different opsins and signaling pathways, or for different types of photoreceptors in the same eye, such as the melanopsin-expressing retinal ganglion cells that coexist with ciliary rods and cones in vertebrate eyes (Lucas, 2013). This suggests that the major classes of opsins shared by metazoans reflect early divergence of photoreceptive tasks.

Obviously, better spatial resolution (an image of more numerous pixels) improves the ability to orient in the environment, but for most kinds of visual orientation, fine spatial details are of little importance. Comparatively low resolution is sufficient for orientation in relation to visible structures or landmarks (Blevins and Johnsen, 2004; Nilsson et al., 2005; Garm et al., 2007, 2011; Garm and Nilsson, 2014; Nilsson and Bok, 2017; Kirwan et al., 2018; Kirwan and Nilsson, 2019; Sumner-Rooney et al., 2020; Ljungholm and Nilsson, 2021). Any level of spatial resolution can help the animals keep a straight path, find a burrow or shelter (or the way out of one), and it can help find a clear path without collisions. The visual flow field will also provide information about self-motion and the distance to surfaces and structures in the environment (Scholtyssek et al., 2014). The use of global cues, such as the direction of the sun, will allow compass orientation, and learned snapshots can support efficient homing (see Grob et al. (2021) for an excellent classification of orientation behaviors).

The information required for visually guided orientation is not so much the spatial structures themselves as it is their motion. The visual flow-field, looming and motion of spatial structures, are essential for guiding locomotion or any other action. It is the changes over time that carry information about what is going on and provides feedback to motor control systems. Spatial vision and motion vision would thus have evolved in close synchrony because one is largely pointless without the other.

Visual orientation may be enhanced by color vision or polarization vision to generate contrasts, but the eyes need not be large or optically sophisticated to provide sufficient resolution for orientation (Nilsson, 2020, 2021). Eyes built to provide spatial resolution are found in about one third of all animal phyla, and in most cases the eyes are small, less than 0.5 mm (Land and Nilsson, 2012; Nilsson, 2021). These eyes are most certainly used to actively guide locomotion toward suitable habitats and to find optimal positions. However, visually guided orientation also requires abilities to assess the environment. Vision can thus be divided into “active vision,” i.e., vision used directly to control actions in a closed feedback loop (see and act) and vision used to “assess the environment” for decisions within a wide time frame (Goodale and Milner, 1992). Visually guided locomotion toward a better place must be preceded by a visual assessment resulting in a decision to abandon the current place. Prior to action, and as part of the assessment, there may also be identification of a direction toward a potentially better habitat.

Visual assessment may also contribute substantially to an even more important function: the choice of suitable behaviors. From their complete behavioral repertoire, all animals must continuously select currently suitable behaviors. In a highly species-specific manner, different behaviors are displayed at different times of day, in different seasons, and in different environments. By visually reading the environment, animals may thus get input for setting a behavioral state (mood-setting) that will make them prone to engage in some behaviors but not in others (Berman et al., 2016; Gurarie et al., 2016; Mahoney and Young, 2017; Nässel et al., 2019). The behavioral state thus acts as a master control of behavior and may additionally adjust the animal’s physiology to prepare it for specific behaviors. These are essential but sadly neglected visual roles.

The types of visual information used for active vision and for visual assessment differ. Active vision is largely based on the movement of edges, structures, and textures. In contrast, information on the environment and its current state is contained more in the general background, its intensity distribution, and spectral distribution. Because this requires only low spatial and temporal resolution, the information can be carried by relatively small populations of slow large-field neurons. The fact that such neurons have not received much attention probably contributes to our poor understanding of vision involved in assessment tasks.

Based on evolutionary reasoning and the moderate demands on spatial resolution, I have previously used the term “low-resolution vision” to classify orientation tasks and other visual tasks that do not require discrimination of objects (Nilsson, 2009, 2013, 2020, 2021; Land and Nilsson, 2012; Nilsson and Bok, 2017). This term is somewhat misleading because visual orientation, and corresponding visual assessment of the environment, may very well rely on higher spatial frequencies in animals with good eyes. Since the original reason that true vision evolved was to serve orientation in the habitat, I here introduce the term *ancient vision* (non-object-based visual tasks), to distinguish it from all visual roles that involve object discrimination (Figure 1). Orientation in the habitat to find suitable locations is the core of ancient vision but it also includes basic visual functions such as measurement of self-motion from optic flow, finding free path, obstacle avoidance, and navigation in relation to landmarks and celestial cues.

## Object Vision Opens a New World

With gradually improved spatial resolution, other animals could in principle be detected at distances beyond the immediate vicinity. However, this is not a trivial extension of visual roles. To detect another animal requires that it can be distinguished as an object separate from the background (image segmentation). It further requires that the object can be identified because the world is full of different types of objects that have to be interacted with in very different ways. A prey, a predator, a conspecific, or an inanimate object all call for very different responses. Apart from a reasonably good spatial resolution, visually guided interaction with other animals thus requires image segmentation to isolate objects, as well as object identification and classification. On top of this, novel neural circuits for interactions with objects, with new motor patterns, would also have to evolve, presumably by duplication and modification of pre-existing circuits for orientation in the inanimate world.

How could such a complex set of traits evolve? It seems challenging to find a gradual evolutionary path where not all new traits had to appear simultaneously to make sense. An attractive possibility is that object vision arose from the ability to recognize a shelter, to help find previously used refuges. This is non-object-based orientation and part of ancient vision, but it requires recognition of a learned spatial pattern and comes a long way toward the visual processing required for object recognition. There may be other orientation tasks that involve memory of spatial patterns, which also offer a smooth transition from non-object-based to object-based vision, making the evolutionary transition less challenging.

Advanced visual processing in combination with high spatial resolution (large pixel numbers) also makes for large and energetically expensive brains (Land and Nilsson, 2012). It is thus no wonder that object-based vision is an exclusive feature which has been fully exploited only in three animal groups: the vertebrates, the arthropods, and the cephalopods (octopus, squid, and cuttlefish). Among the arthropods, object-based vision may have evolved independently more than once, as indicated by compound eyes in insects and crustaceans and non-homologous camera-type eyes in spiders [their principal eyes (Nilsson, 2020)]. In arthropods, the situation is complicated by visual systems comprising both lateral and median eyes. In insects and crustaceans the median eyes (dorsal ocelli and nauplius eyes) are not know to be implicated in object discrimination tasks, leaving the lateral compound eyes responsible for their object vision. However, the compound eyes are certainly also involved in orientation and other tasks of ancient vision.

Object vision may be both expensive and neurally demanding, but the benefits are enormous. The ability to detect and identify other animals and food items at some distance offers a tremendous potential for new and advanced behaviors. One of these, visually guided predation, must have led to a major ecological turnover pushing for good vision and swift locomotion in both prey and predator species. The fact that the maximum distance for object recognition is closely related to spatial resolution [visual acuity (Nilsson et al., 2014)], suggests that there were evolutionary arms races in visual performance between competing predators and their prey species, and this would have rapidly led to large eyes and high-performance vision. Other evolutionary responses to the new threat would have been development of protective armor or deep burrowing life-styles to escape visually guided predators. The fossil record reveals that all these features appeared rapidly during the Cambrian explosion, some 540 million years ago. It seems possible, and even likely, that the evolution of object vision and visually guided predation rapidly drove this evolution and generated essentially all modern and swiftly mobile animals from pre-existing small, soft and slow animals.

A huge range of novel visually guided behaviors were made possible by object vision. Apart from its obvious use for catching prey, and for visually guided escape from predators, object vision allows for recognition of conspecifics, visual communication and other visually guided interactions for reproductive and social purposes. Further examples are detection and manipulation of food items.

I analogy with ancient visual roles, object vision can be divided into active tasks of visuomotor control and assessment tasks with both short- and long-term impacts on behavior. Visual perception, which is a central concept in human vision, is largely equivalent to object-based visual assessment. There is no reason to doubt that other vertebrates, arthropods and cephalopods are also capable of visual perception. In contrast to non-object based visual assessment, which informs about habitat quality and current conditions, perception provides information on the current situation and allows for the planning of actions. This too is a major advance from just ancient vision.

Based on human studies, Goodale and Milner (1992) made the important principal distinction between active object-vision and perception. Interestingly, observations of patients with acquired neurological dysfunction suggested that perception is a conscious process whereas active visual tasks in general are not [see also Goodale (2014)]. Whether this also applies to other species among vertebrates, arthropods and cephalopods is difficult to test but fascinating food for speculation.

In the animal groups where object vision has evolved (vertebrates, arthropods, and cephalopods) the ancient visual tasks remain as important as they were before object vision evolved. In fact, ancient vision may exploit the higher resolution required for object vision, resulting in improve performance of general orientation, navigation, path finding, obstacle avoidance, and measurements of self-motion. Large eyes, extensive retinas, and high pixel numbers in neural processing are expensive requirements for object vision (Land and Nilsson, 2012). These costs are offset by the enormous advantages endowed by visual discrimination of objects. Neural circuits for early visual processing and motion detection may be shared by object vision and active ancient vision, making the latter benefit from a major performance boost without much extra cost. Introduction of object vision can thus be expected to result also in major improvements of ancient vision (Figure 1).

## Discussion

The roles that vision serve can be neatly divided into ancient vision and object vision, where the former must have evolved before the latter. The distinction between active visual tasks for immediate feed-back control of action on the one hand, and assessment tasks, with potentially long time-constants, on the other, applies to both ancient vision and object vision. It is impossible to say whether active vision or assessment vision evolved first. In many ways, the two modes depend on one another: habitat orientation and interaction with objects would normally require a previous assessment of the environment. Likewise, visual assessments make sense only if they lead to behavioral decisions and these may in turn result in visually guided actions.

The overall evolutionary sequence from non-directional photoreception, *via* directional photoreception and ancient vision, to object vision (Figure 1), represents a natural increase in the amount of sensory information, eye complexity, and neural complexity. Each step preadapts the sensory system for the next step, implying that it is practically inconceivable to jump any of the steps. The numerous invertebrates at intermediate stages in the sequence offers strong support that the four-step model of visual evolution is not only general but has been followed independently in different animal phyla (Nilsson, 2020). The only groups that are documented to have object vision are vertebrates, arthropods and cephalopods. Many more groups, such as gastropod mollusks, polychetes and jellyfish have stopped at ancient vision, but a few of these groups contain examples that may qualify for object vision (conchs, heteropod snails and alciopid polychetes [see Land, 1981)]. Numerous taxonomic groups have stopped at directional photoreception but it is rare to possess only non-directional photoreception.

There are a few notable exceptions to the general evolutionary sequence of vision. These exceptions are found in sessile or very slow-moving invertebrates where the original eyes for ancient vision have been reduced and new, molecularly different, eyes or dispersed photoreceptors have evolved in novel locations to warn the animal of approaching danger (Bok et al., 2016, 2017; Nilsson, 2021). This has happened in fan worms, bivalves, and chitons, with alarm photoreceptors on the tentacular crown in fan worms, along the mantel edge in bivalves (Morton, 2008; Audino et al., 2020) and over the dorsal shell plates in chitons (Kingston et al., 2018). In fan worms and bivalves, the molecular profile indicates that these photoreceptors and eyes evolved directly from dermal shadow receptors (non-directional photoreception) (Del Pilar Gomez and Nasi, 2000; Bok et al., 2017).

In the past, the evolution of vision has been investigated *via* studies of developmental genes (Arendt, 2003), opsin sequences (Porter et al., 2012; Ramirez et al., 2016) or eye structure (Nilsson, 2009). These studies have added valuable insight into the evolution of vision, but it is important to note that the driving force does not lie at any of these organizational levels. It is the fitness gained by more efficient behaviors that have been the driving force behind the evolution of vision. It should also be emphasized that eyes, brains, and the motile body must evolve in concert to produce a fitness gain through more advanced vision.

## Data Availability Statement

The original contributions presented in the study are included in the article/supplementary material, further inquiries can be directed to the corresponding author.

## Author Contributions

The author confirms being the sole contributor of this work and has approved it for publication.

## Conflict of Interest

The author declares that the research was conducted in the absence of any commercial or financial relationships that could be construed as a potential conflict of interest.

## Publisher’s Note

All claims expressed in this article are solely those of the authors and do not necessarily represent those of their affiliated organizations, or those of the publisher, the editors and the reviewers. Any product that may be evaluated in this article, or claim that may be made by its manufacturer, is not guaranteed or endorsed by the publisher.
